# Short and sweet

**DOI:** 10.7554/eLife.55220

**Published:** 2020-02-25

**Authors:** Coleen T Murphy

**Affiliations:** 1 Department of Molecular Biology, Princeton UniversityPrincetonUnited States; 2 LSI Genomics, Princeton UniversityPrincetonUnited States

**Keywords:** dauer formation, alternative splicing, insulin sensitivity, DAF-2, aging, longevity, *C. elegans*

## Abstract

A truncated version of the only insulin receptor in *C. elegans* has been discovered.

**Related research article** Martinez BA, Rodrigues PR, Nuñez Medina RM, Mondal P, Harrison NJ, Lone MA, Webster A, Gurkar AU, Grill B, Gill MS. 2020. An alternatively spliced, non-signaling insulin receptor modulates insulin sensitivity via insulin peptide sequestration in *C. elegans*. *eLife* **9**:e49917. doi: 10.7554/eLife.49917

It would be hard to overstate the importance of a receptor called DAF-2 to our understanding of aging and longevity. Almost 30 years ago it was discovered that loss of the *daf-2* gene doubles lifespan in the worm *C. elegans* (Kenyon et al., 1993), and a few years later it was reported that DAF-2 is the only insulin/IGF-1-like receptor in *C. elegans* (Kimura et al., 1997). These findings led to an explosion of research into aging and longevity, revealing an intricate insulin signaling pathway that coordinates the sensing of nutrient levels with the regulation of age-related decline. In particular, it was found that reduced insulin signaling extends lifespan and increases stress resistance in flies and mice (Clancy et al., 2001; Holzenberger et al., 2003). Moreover, mutations in some of the genes associated with this pathway were found in centenarians (Suh et al., 2008). And in worms it became clear that, in addition to longevity and age-related declines, DAF-2 is involved in the regulation of a wide range of biological processes, including development, reproduction, memory, and stress responses.

DAF-2 was originally discovered for its role in controlling the dauer stage – an alternative stage of development in which a larva goes into a type of stasis to help it survive harsh conditions (Riddle et al., 1981). A lack of DAF-2 causes *C. elegans* to enter dauer, as does a lack of a number of other kinases (Paradis and Ruvkun, 1998). An ongoing mystery is why *C. elegans* has just a single gene for an insulin receptor despite having 40 different insulin-like peptides (Pierce et al., 2001). Some of these peptides are agonists (that is, they activate the receptor) and others are antagonists (they inhibit the receptor).

Given three decades of extensive research into the insulin signaling pathway in *C. elegans*, it would be shocking to find new functions for DAF-2 at this point. However, in a new paper in *eLife*, Matthew Gill of the Scripps Research Institute and colleagues – including Bryan Martinez and Pedro Reis Rodrigues as joint first authors – report evidence for such a shock: the gene for DAF-2 can also express another, truncated isoform of this protein as a result of alternative splicing (Martinez et al., 2020). The truncated version, which is called DAF-2B, can still form dimers but, unlike the full-length version, it is not expected to be able to span the membrane: this suggests that the truncated form could be secreted.

Truncated insulin receptors that have extracellular ligand-binding domains, but lack intracellular signaling domains, have been reported in both *Drosophila *and mammals, and are known to sequester insulin peptides. However, in these cases the full-length receptors and the truncated receptors are expressed from separate genes. Martinez et al. found that although DAF-2B was expressed in neuronal cells, it accumulated in cells called coelomocytes (macrophage-like cells that attack invading organisms such as bacteria and viruses). These results suggest that DAF-2B can indeed be secreted, rather than being retained in the neurons in which it is expressed and spliced.

But what does this shortened form of DAF-2 do? The best-characterized functions of the insulin signaling pathway are dauer formation and lifespan regulation, so Martinez et al. used these phenotypes to study DAF-2B. They found that overexpressing DAF-2B increased dauer formation, slowed dauer exit, and increased lifespan, whereas a lack of DAF-2B had the opposite effect. Basically, the data suggest that the function of DAF-2B is essentially the opposite of the function of DAF-2.

Martinez et al. also explored the interactions between DAF-2B and insulin-like peptides that were either agonists or antagonists. Overexpression of two peptides that are agonists (DAF-28 and INS-6) reduced the dauer-promoting effects of DAF-2B. Conversely, the overexpression of a peptide that is an antagonist (INS-18) would be expected to promote dauer, but this effect was blunted when DAF-2B was also overexpressed. Additionally, the researchers found that a point mutation in the proposed insulin-binding domain resulted in a form of DAF-2B that exhibited reduced dauer formation. Together, these results suggest that DAF-2B binds and may sequester insulin-like peptides, and/or form dimers with DAF-2.

Of course, mysteries remain. Given that worms have dozens of insulin-like peptides (Pierce et al., 2001), which of these bind to DAF-2B, and under what circumstances? And if DAF-2B is secreted, why does it matter where it is expressed, unless there are highly localized interactions? Finally, the mechanism by which DAF-2B acts and its dimerization state is not entirely understood.

The discovery of the truncated version of DAF-2, and the fact that it essentially works in opposition to the full-length version, raises new questions and will change how we think about DAF-2's role in insulin signaling regulation of aging and longevity.

## References

[bib1] Clancy DJ, Gems D, Harshman LG, Oldham S, Stocker H, Hafen E, Leevers SJ, Partridge L (2001). Extension of life-span by loss of CHICO, a *Drosophila* insulin receptor substrate protein. Science.

[bib2] Holzenberger M, Dupont J, Ducos B, Leneuve P, Géloën A, Even PC, Cervera P, Le Bouc Y (2003). IGF-1 receptor regulates lifespan and resistance to oxidative stress in mice. Nature.

[bib3] Kenyon C, Chang J, Gensch E, Rudner A, Tabtiang R (1993). A *C. elegans* mutant that lives twice as long as wild type. Nature.

[bib4] Kimura KD, Tissenbaum HA, Liu Y, Ruvkun G (1997). *daf-2*, an insulin receptor-like gene that regulates longevity and diapause in *Caenorhabditis elegans*. Science.

[bib5] Martinez BA, Rodrigues PR, Nuñez Medina RM, Mondal P, Harrison NJ, Lone MA, Webster A, Gurkar AU, Grill B, Gill MS (2020). An alternatively spliced, non-signaling insulin receptor modulates insulin sensitivity via insulin peptide sequestration in *C. elegans*. eLife.

[bib6] Paradis S, Ruvkun G (1998). *Caenorhabditis elegans* akt/PKB transduces insulin receptor-like signals from AGE-1 PI3 kinase to the DAF-16 transcription factor. Genes & Development.

[bib7] Pierce SB, Costa M, Wisotzkey R, Devadhar S, Homburger SA, Buchman AR, Ferguson KC, Heller J, Platt DM, Pasquinelli AA, Liu LX, Doberstein SK, Ruvkun G (2001). Regulation of DAF-2 receptor signaling by human insulin and *ins-1*, a member of the unusually large and diverse *C. elegans* insulin gene family. Genes & Development.

[bib8] Riddle DL, Swanson MM, Albert PS (1981). Interacting genes in nematode dauer larva formation. Nature.

[bib9] Suh Y, Atzmon G, Cho MO, Hwang D, Liu B, Leahy DJ, Barzilai N, Cohen P (2008). Functionally significant insulin-like growth factor I receptor mutations in centenarians. PNAS.

